# Supplementary material for: Functional ultrasound localization microscopy reveals brain-wide neurovascular activity on a microscopic scale
Source: Nat Methods. 2022 Aug 4;19(8):1004–12. doi: 10.1038/s41592-022-01549-5 (PMC9352591; doi:10.1038/s41592-022-01549-5)
Supplement: Supplementary file 1 — Supplementary Figs. 1–5 [file 41592_2022_1549_MOESM1_ESM.pdf]

nature portfolio

https://doi.org/10.1038/s41592-022-01549-5

Supplementary information

Functional ultrasound localization microscopy reveals brain-wide neurovascular activity on a microscopic scale

In the format provided by the authors and unedited

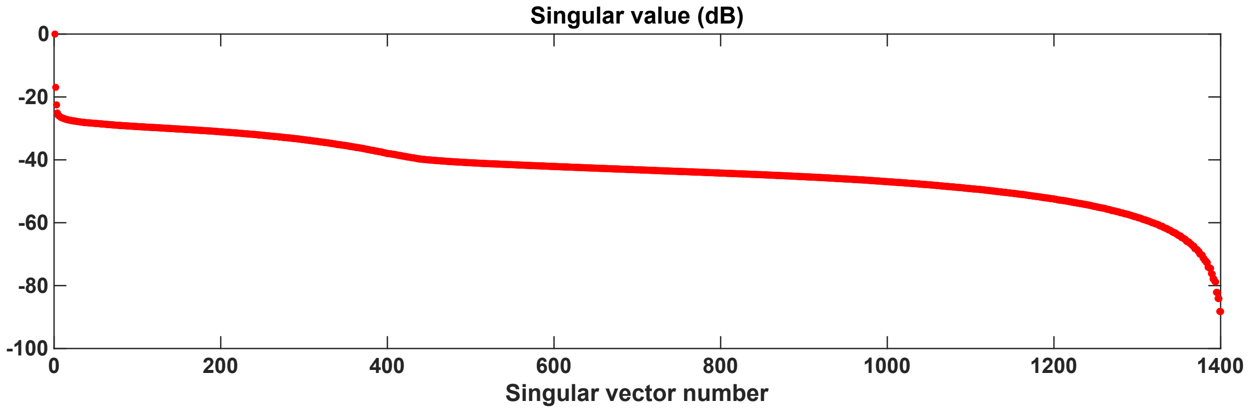

Supplementary figure 1 : Singular values distribution in dB after SVD decomposition of raw temporal ULM data (whisker stimulation). Single micrograph.

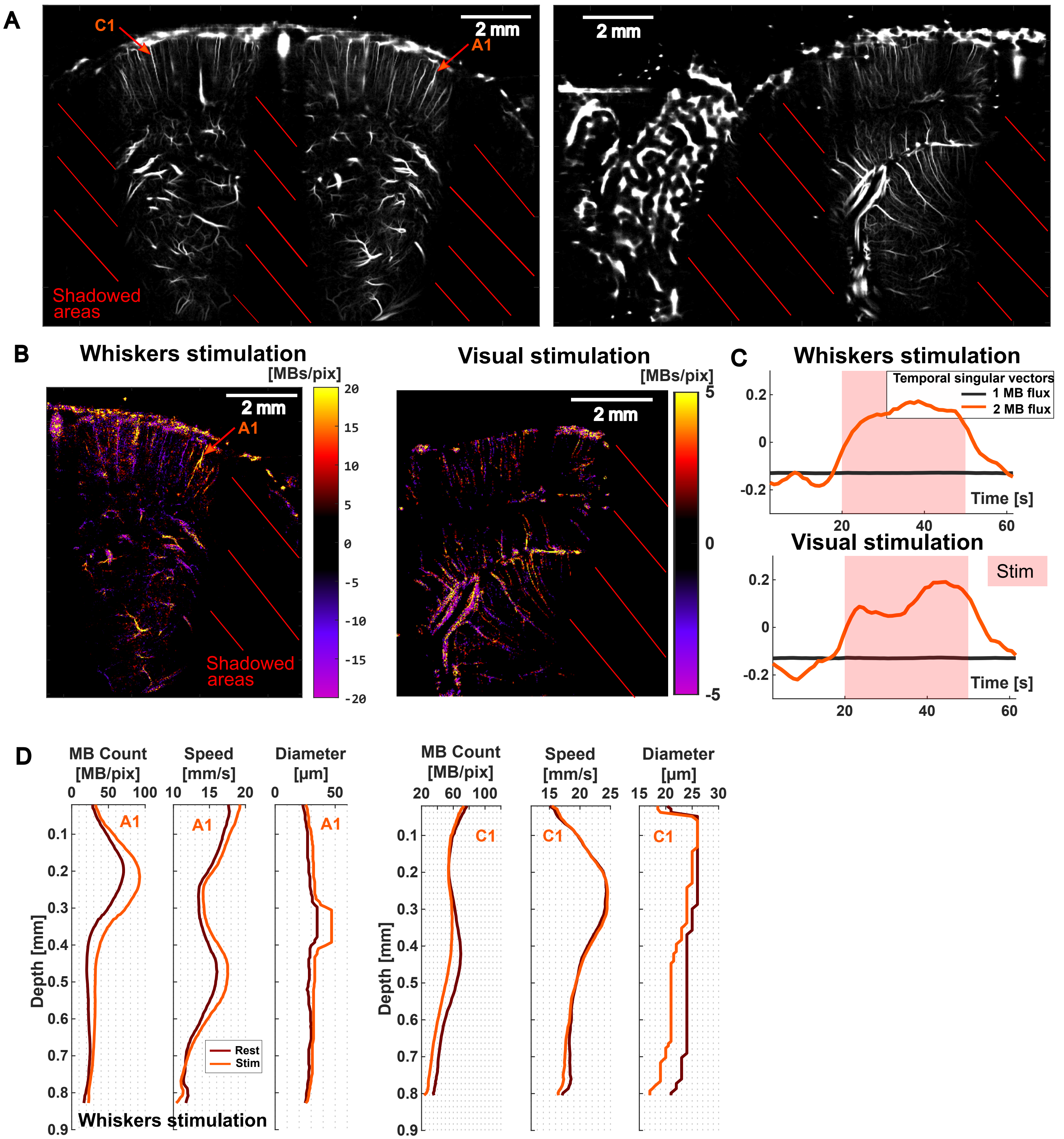

Supplementary figure 2: Transcranial fULM. (**A**) ULM maps of a rat brain using transcranial imaging in a plane for whiskers stimulation (left) and visual stimulation (right). The red lines point out shadowed areas caused by a stripe artifact (aberrations due to a curvature where major pial vessels run in the inner surface of the skull). (**B**) SVD analysis on the MB flux signal in the two planes shown in (A). Spatial singular vectors corresponding to functional hyperaemia: whisker stimulation (left) and visual stimulation (right). (**C**) Corresponding temporal singular vectors. (**D**) MB count, Speed and diameter quantifications for rest and stimulation periods for the blood vessel A1 in the activated barrel and C1 in a control area. Their location is shown in (A) and (B). (A-D) Single micrographs.

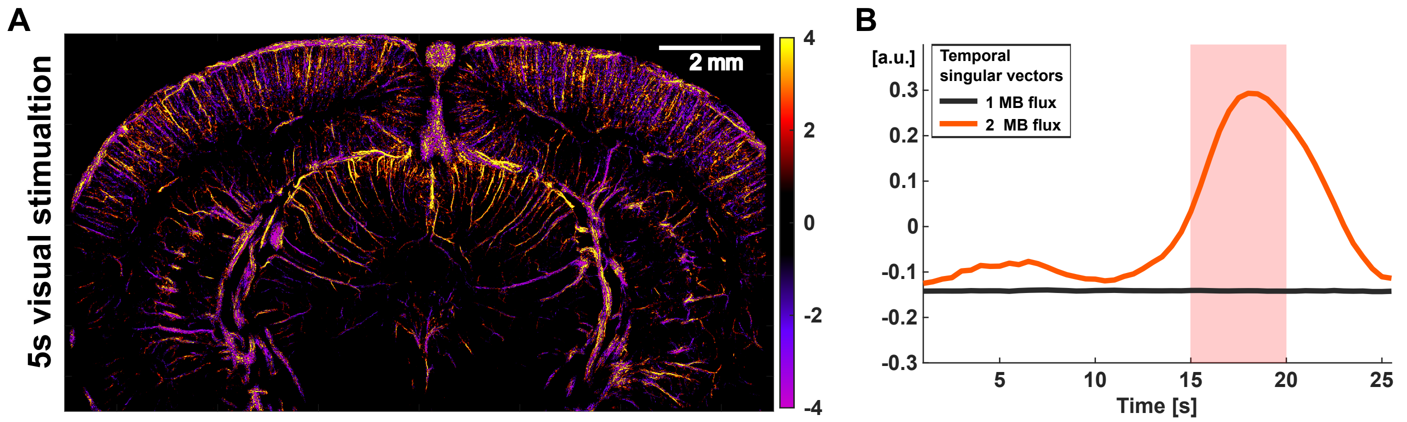

Supplementary figure 3: Short stimulation paradigm. 5 seconds visual stimulation. Data were accumulated in 2 seconds (window length=2s, step = 500ms) to create one ULM image but the number of repetitions was increased to N=60. Results show the spatial singular vector corresponding to stimulation (**A**) and the first SVD temporal singular vectors (**B**).
(A-B) Single micrographs.

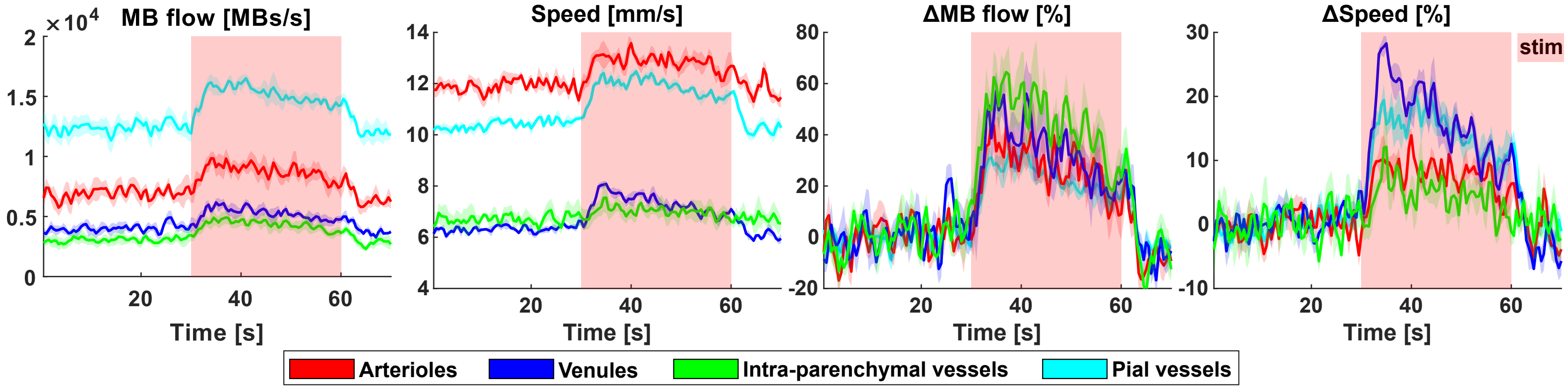

Supplementary figure 4: Vascular compartment analysis with a finer temporal resolution. Same analysis as in Fig. 2C but the sliding window used for ULM temporal data construction is now 1 second with a 0.5 second step (instead of 5 seconds with a 1 second step in Fig. 2C). Mean MB flow and speed (± SE) from N=4 time courses obtained on 10 stimulations each, either expressed as absolute value for each type of blood vessels (two left panels), or as relative to baseline (two right panels).

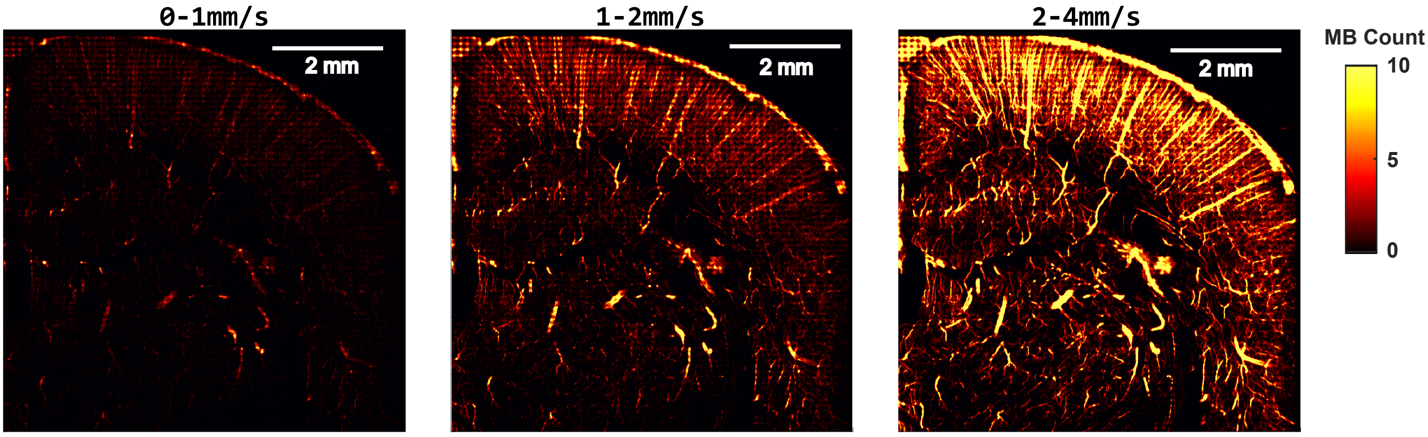

Supplementary figure 5: Sensitivity to slow flow. MB count ULM maps computed using microbubbles flowing in three different velocity intervals: 0-1 mm/s, 1-2 mm/s and 2-4 mm/s. N=8 experiments.
