# Supplementary material for: Functional ultrasound localization microscopy reveals brain-wide neurovascular activity on a microscopic scale
Source: Nat Methods. 2022 Aug 4;19(8):1004–12. doi: 10.1038/s41592-022-01549-5 (PMC9352591; doi:10.1038/s41592-022-01549-5)
Supplement: Supplementary file 2 — Reporting Summary [file 41592_2022_1549_MOESM2_ESM.pdf]

nature portfolio

Corresponding author(s): Mickael Tanter

Last updated by author(s): Jan 17, 2022

Reporting Summary

Nature Portfolio wishes to improve the reproducibility of the work that we publish. This form provides structure for consistency and transparency in reporting. For further information on Nature Portfolio policies, see our Editorial Policies and the Editorial Policy Checklist.

Statistics

For all statistical analyses, confirm that the following items are present in the figure legend, table legend, main text, or Methods section.

n/a	Confirmed	
☐	☒	The exact sample size (*n*) for each experimental group/condition, given as a discrete number and unit of measurement
☐	☒	A statement on whether measurements were taken from distinct samples or whether the same sample was measured repeatedly
☐	☒	The statistical test(s) used AND whether they are one- or two-sided<br>*Only common tests should be described solely by name; describe more complex techniques in the Methods section.*
☐	☒	A description of all covariates tested
☐	☒	A description of any assumptions or corrections, such as tests of normality and adjustment for multiple comparisons
☐	☒	A full description of the statistical parameters including central tendency (e.g. means) or other basic estimates (e.g. regression coefficient) AND variation (e.g. standard deviation) or associated estimates of uncertainty (e.g. confidence intervals)
☐	☒	For null hypothesis testing, the test statistic (e.g. *F*, *t*, *r*) with confidence intervals, effect sizes, degrees of freedom and *P* value noted<br>*Give P values as exact values whenever suitable.*
☒	☐	For Bayesian analysis, information on the choice of priors and Markov chain Monte Carlo settings
☒	☐	For hierarchical and complex designs, identification of the appropriate level for tests and full reporting of outcomes
☒	☐	Estimates of effect sizes (e.g. Cohen's *d*, Pearson's *r*), indicating how they were calculated

Our web collection on statistics for biologists contains articles on many of the points above.

Software and code

Policy information about availability of computer code

Data collection	Raw Ultrasound data was acquired using a linear ultrasound probe driven by a prototype ultrasonic ultrafast neuroimager (Iconeus, France) and the with Neuroscan live acquisition software (version 1.3, Iconeus, Paris, France and Inserm Accelerator of Technological Research in Biomedical Ultrasound, Paris, France).
Data analysis	Data analysis were performed on Matlab R2020b (MathWorks, Cambridge, MA, USA).<br>Home-made Matlab codes were used for the ULM algorithms: SVD filtering of tissue signal (according to Demené, C. et al. Spatiotemporal Clutter Filtering of Ultrafast Ultrasound Data Highly Increases Doppler and fUltrasound Sensitivity. IEEE Transactions on Medical Imaging 34, 2271–2285 (2015)), localization of the microbubbles.<br>The ULM algorithm includes a vesselness filtering available on Mathworks file exchange (2D implementation available on Mathworks file exchange, ©Dirk-Jan Kroon2009, and © Tim Jerman, 2017) and a tracking algorith, simpletracker.m available on Mathworks ©Jean-Yves Tinevez, wrapping matlab munkres algorithm implementation of ©Yi Cao 2009.Home-made Matlab codes were used for the ULM data analysis and the statistical analysis.<br>Superresolution movies were obtained using a 3D software for visualization (Houdini, 17.5.360, SideFX, Toronto, Canada).<br>Matlab codes for the reading of ULM data are provided on the Zenodo repository website at: 10.5281/zenodo.6109803<br>Low level acquisition and Processing codes of the raw data used for the collection of ULM data are protected by INSERM and can only be shared upon request, with the written agreement of INSERM.

For manuscripts utilizing custom algorithms or software that are central to the research but not yet described in published literature, software must be made available to editors and reviewers. We strongly encourage code deposition in a community repository (e.g. GitHub). See the Nature Portfolio guidelines for submitting code & software for further information.

Data

Policy information about availability of data

All manuscripts must include a data availability statement. This statement should provide the following information, where applicable:

- Accession codes, unique identifiers, or web links for publicly available datasets
- A description of any restrictions on data availability
- For clinical datasets or third party data, please ensure that the statement adheres to our policy

ULM Data for data analysis are available on the zenodo repository website at: 10.5281/zenodo.6109803

Field-specific reporting

Please select the one below that is the best fit for your research. If you are not sure, read the appropriate sections before making your selection.

☒ Life sciences ☐ Behavioural & social sciences ☐ Ecological, evolutionary & environmental sciences

For a reference copy of the document with all sections, see nature.com/documents/nr-reporting-summary-flat.pdf

Life sciences study design

All studies must disclose on these points even when the disclosure is negative.

Sample size	No sample size calculation was performed as the proof of concept of of functional Ultrasound Localization Microscopy was successful in all N=10 animals. Sample size was chosen by considering 100% success rate in detecting and mapping a functional brain activity after N>=3 animals was sufficient. N=4 animals were used for whiskers stimualtions. N=3 animals were used for visual stimulations. N=3 additional animals were used as examples for different experimental conditions testing.
Data exclusions	Preliminary experiments (N=4 animals) were used for protocol optimization (mainly microbubble injection optimization). All animals involved after that were successful and used in the study, no data exclusion.
Replication	For this proof of concept experiments, all attempts at replication were successful. Processing steps were performed the same way for all animals, following what is described in the manuscript.
Randomization	We only used naive animals. Among them, the animals were chosen randomly. No group comparison was performed in this proof of concept publication.
Blinding	Blinding was not relevant as ou study consists in a technical proof of concept study, and does not involve comparison between groups.

Reporting for specific materials, systems and methods

We require information from authors about some types of materials, experimental systems and methods used in many studies. Here, indicate whether each material, system or method listed is relevant to your study. If you are not sure if a list item applies to your research, read the appropriate section before selecting a response.

Materials & experimental systems

n/a	Involved in the study	
☒	☐	Antibodies
☒	☐	Eukaryotic cell lines
☒	☐	Palaeontology and archaeology
☐	☒	Animals and other organisms
☒	☐	Human research participants
☒	☐	Clinical data
☒	☐	Dual use research of concern

Methods

n/a	Involved in the study	
☒	☐	ChIP-seq
☒	☐	Flow cytometry
☒	☐	MRI-based neuroimaging

Animals and other organisms

Policy information about studies involving animals; ARRIVE guidelines recommended for reporting animal research

Laboratory animals	Experiments were performed on N=10 male Sprague–Dawley rats (Janvier Labs; Le Genest St Isle, France), weighing 200-300g (Age 7-9 weeks)
Wild animals	The study did not involve wild animals.

Field-collected samples	The study did not involve samples collected from the field.
Ethics oversight	All experiments were performed in agreement with the European Community Council Directive of September 22, 2010 (010/63/UE) and the local ethics committee (Comité d'éthique en matière d'expérimentation animale N°59, 'Paris Centre et Sud', project #2017-23)

Note that full information on the approval of the study protocol must also be provided in the manuscript.
